# Neighborhood-Regularized Matrix Factorization for lncRNA–Disease Association Identification

**DOI:** 10.3390/ijms26094283

**Published:** 2025-04-30

**Authors:** Jihwan Ha, Kwangsu Kim

**Affiliations:** 1 Major of Big Data Convergence, Division of Data Information Science, Pukyong National University, Busan 48513, Republic of Korea; jhha@pknu.ac.kr; 2 Department of Scientific Computing, Pukyong National University, Busan 48513, Republic of Korea

**Keywords:** machine learning, matrix factorization, lncRNA, disease, lncRNA–disease association

## Abstract

Long non-coding RNAs (lncRNAs) have been shown to be integral in a variety of biological processes and significantly influence the progression of several human diseases. Their involvement in disease mechanisms makes them crucial targets for research in disease biomarker identification. Understanding the intricate relationships between lncRNAs and diseases can offer valuable insights for advancing diagnostic, prognostic and therapeutic strategies. In light of this, we propose a recommendation-system-based model utilizing matrix factorization with disease neighborhood regularization to effectively infer disease-related lncRNAs (NRMFLDA). This approach leverages the power of matrix factorization techniques while incorporating disease neighborhood regularization to enhance the accuracy and reliability of lncRNA–disease association predictions. Consequently, NRMFLDA exhibits outstanding performance, achieving AUC scores of 0.9143 and 0.8993 in both leave-one-out and five-fold cross-validation, surpassing the performance of four previous models. This demonstrates its effectiveness and robustness in accurately predicting disease-related lncRNAs. We believe that NRMFLDA will not only provide innovative approaches for uncovering lncRNA–disease associations but also contribute significantly to the identification of novel biomarkers for various diseases, thereby advancing diagnostic and therapeutic strategies.

## 1. Introduction

Genomic studies reveal that less than 2% of the human genome is involved in coding for proteins, while the remaining 98% does not directly translate into protein products [1,2]. The portion of the genome that does not code for proteins often gives rise to non-coding RNAs [3]. For many years, these non-coding RNAs were thought to be irrelevant byproducts of transcription, often dismissed as mere “transcriptional noise” [4,5]. However, emerging research is beginning to shed light on their significant roles in regulating various biological processes, suggesting they may be far more important than previously assumed. Recent studies have revealed that long non-coding RNAs (lncRNAs), which are typically over 200 nucleotides in length, are crucial regulators in a wide array of biological functions. These include processes such as cell differentiation, immune response modulation, transcriptional and translational regulation and cell proliferation, among others [6,7,8,9,10,11,12,13,14]. Unlike their shorter counterparts, lncRNAs have emerged as key players in controlling gene expression and maintaining cellular homeostasis, highlighting their importance beyond just structural components of the genome. Furthermore, numerous studies have shown that the maturation and improper regulation of lncRNAs can contribute to the development of complex human diseases. For example, the expression of HOX antisense intergenic RNA (HOTAIR), a well-known lncRNA, has been linked to the onset of several cancer types, including breast, colon and liver cancers [15,16]. This highlights the potential of lncRNAs as key factors in disease progression, offering new insights into the molecular mechanisms underlying these conditions. Additionally, the breast cancer anti-estrogen resistance 4 (BCAR4) lncRNA has been identified as an oncogene in breast cancer and is also found to be upregulated in colon cancer tissues [17,18,19]. As a result, uncovering the associations between lncRNAs and diseases has become a critical endeavor, particularly for the identification of potential biomarkers and for gaining a deeper understanding of the molecular mechanisms driving complex human diseases.

Compared to traditional wet-lab experiments, computational approaches offer significant advantages in terms of time efficiency and cost effectiveness when it comes to predicting disease-associated long non-coding RNAs (lncRNAs). These methods can quickly process large datasets, making them ideal for handling the complexity of biological systems. As a result, various computational models have been developed to uncover new associations between lncRNAs and diseases, helping accelerate the discovery of potential biomarkers or therapeutic targets without the need for extensive experimental work. Chen et al. introduced a computational approach grounded in the well-established hypothesis that lncRNAs with similar functions are associated with diseases exhibiting comparable phenotypes [20]. In their work, the authors proposed a model named HGLDA, which leverages lncRNA–disease association datasets alongside lncRNA–miRNA interaction data [21]. HGLDA has been demonstrated to be an efficient model, especially in identifying novel disease-related lncRNAs without relying on known positive lncRNA–disease associations. Additionally, Chen et al. developed another computational model, IRWRLDA, for predicting lncRNA–disease associations. This model integrates multiple similarity measures, including lncRNA expression patterns, disease semantic relationships and functional lncRNA similarities [22]. Yu et al. proposed a heterogeneous data-based framework that excels in identifying lncRNA–disease associations by utilizing a bi-random walk algorithm [23]. In another study, Gu et al. introduced GrwLDA, a network-based method that employs a random walk algorithm to predict lncRNA–disease associations. Notably, this model is applicable to lncRNAs with no prior known disease associations [24]. Zou et al. focused on prioritizing disease-associated lncRNAs by constructing diverse heterogeneous networks, including lncRNA–lncRNA cross-talk networks, disease–disease similarity networks and lncRNA–disease association networks [25]. These approaches highlight the importance of incorporating multiple layers of data and network structures to enhance the accuracy and applicability of lncRNA–disease association predictions. Zhang et al. proposed LDAGM, a method for predicting lncRNA–disease associations by integrating functional and semantic similarities into a multi-view heterogeneous network. The approach utilizes a graph convolutional autoencoder for non-linear feature extraction and a multi-layer perceptron with an aggregation layer to enhance prediction performance and stability [26]. Wang et al. proposed ResGCN-A, a novel lncRNA–disease prediction method that integrates attention mechanisms with a residual graph convolutional network. The method combines lncRNA and disease similarities, extracts local features using the residual graph convolutional network and enhances feature weights through the attention mechanism to improve prediction accuracy using an extra-trees classifier [27].

The rapid development of computational technologies has led to the widespread adoption of machine-learning techniques, which have demonstrated remarkable success across various scientific domains [28,29,30,31,32,33,34]. Among these techniques, matrix factorization (MF) has gained significant recognition, particularly in the realm of recommender systems, due to its scalability and high performance. As a result, MF has found applications beyond recommender systems, extending into areas such as bioinformatics [35,36]. For example, Lu et al. developed an MF-based framework to predict lncRNA–disease associations by calculating the Gaussian interaction profile kernel between lncRNAs and diseases [37]. Similarly, Fu et al. introduced a computational model that employs tri-matrix factorization to decompose heterogeneous data into low-rank latent spaces, enabling the identification of novel lncRNA–disease associations (MFLDA) [38]. Xuan et al. proposed a probabilistic matrix factorization approach (PMFILDA) for predicting disease-related lncRNAs, which incorporates networks such as the lncRNA–miRNA association network, the miRNA–disease association network and the lncRNA–disease correlation network. The model further applies the KNN algorithm to uncover new lncRNAs associated with diseases [39]. These advancements highlight the growing potential of matrix factorization methods in the exploration and prediction of complex biological relationships. Lan et al. introduced a computational framework based on a graph attention network, named GANLDA, to predict lncRNAs associated with diseases [40]. In this approach, the graph attention network was utilized to effectively extract features related to both lncRNAs and diseases. Subsequently, a multi-layer perceptron (MLP) was employed to forecast novel lncRNA–disease associations. Peng et al. proposed LDA-VGHB, a framework for predicting lncRNA–disease associations that integrates feature extraction using singular value decomposition and variational graph autoencoder with classification through a heterogeneous Newton boosting machine. They demonstrated that LDA-VGHB outperformed existing methods and models in multiple cross-validation settings, highlighting its potential for identifying lncRNAs linked to complex diseases [41]. Ha et al. proposed EMFLDA, a novel matrix-factorization-based method that incorporates lncRNA expression profiles as weights to identify lncRNA–disease associations. This approach effectively integrates heterogeneous biological datasets, enhancing the model’s ability to detect meaningful associations [42].

In this study, we present a novel neighborhood-regularized matrix factorization framework that works well in predicting novel disease-related lncRNAs (NRMFLDA). The key contributions of the proposed NRMFLDA framework can be outlined as follows: NRMFLDA utilizes matrix factorization, a collaborative filtering approach, to transform known lncRNA–disease associations into a unified latent space, capturing essential latent features of both lncRNAs and diseases. To enhance the representation of this latent space, a disease-specific neighborhood regularization is incorporated, enabling more precise modeling of the underlying relationships. Moreover, the integration of lncRNA expression profiles into the matrix factorization process further strengthens the model’s predictive ability. This integration not only boosts performance but also ensures that the machine-learning model reflects relevant biological mechanisms, thereby bridging computational predictions with biological insights. Consequently, extensive experimental results demonstrate that NRMFLDA achieves superior performance in terms of AUC scores (0.9143, 0.8993) based on leave-one-out cross-validation (LOOCV) and five-fold cross-validation. Also, case studies on human major cancers (gastric, lung and prostate) clearly validate the efficacy and superiority of NRMFLDA.

## 2. Results

### 2.1. Evaluation Metric

To assess the effectiveness of NRMFLDA, we employed various performance metrics, with leave-one-out cross-validation (LOOCV) being a prominent choice for estimating model reliability. LOOCV, a specific form of n-fold cross-validation, ensures that each individual data point serves as the test set exactly once during the validation process. This approach is particularly advantageous when working with limited datasets or when rigorous validation is essential. LOOCV can be divided into two categories: global LOOCV and local LOOCV. In the global variant, all diseases are evaluated collectively, providing a comprehensive view of the model’s performance across multiple conditions. Conversely, local LOOCV focuses exclusively on a single disease, offering insights into the model’s effectiveness in specific scenarios. This dual approach enables a balanced analysis of the model’s generalizability and its disease-specific accuracy. To further illustrate the model’s predictive capabilities under both global and local LOOCV frameworks, receiver operating characteristic (ROC) curves were generated. In these plots, the false positive rate (FPR) is displayed along the *x*-axis, while the true positive rate (TPR), also referred to as sensitivity, is represented on the *y*-axis. Sensitivity and specificity were calculated using the following standard Formulae (1) and (2). Sensitivity (TPR) quantifies the proportion of actual positives correctly identified by the model, while specificity measures the proportion of negatives accurately classified. These metrics collectively provide a robust evaluation of the model’s diagnostic precision, ensuring a reliable assessment of its applicability in practical scenarios.

(1) $$TPR=\frac{TP}{TP+FN}$$

(2) $$FPR=\frac{FP}{FP\;+TN}$$

Furthermore, we incorporated multiple performance indicators, including accuracy (ACC), the Matthews correlation coefficient (MCC) and the area under the precision–recall curve (AUPRC). The precision–recall (PR) curve is defined by recall on the *x*-axis and precision on the *y*-axis, providing a detailed visualization of the model’s ability to balance these two critical aspects. The aforementioned evaluation criteria were computed using specific mathematical formulae, ensuring consistent and reproducible assessments. These metrics offer complementary insights: while accuracy reflects the overall correctness of predictions, MCC provides a balanced measure that accounts for both true and false predictions, particularly in imbalanced datasets. AUPRC, on the other hand, evaluates the trade-off between precision and recall, which is especially valuable in scenarios with skewed class distributions.

(3) $$precision=\frac{TP}{TP\;+\;FP}$$

(4) $$recall=\frac{TP}{TP+FN}$$

(5) $$ACC=\frac{TP+TN}{TP+TN+FP+FN}$$

(6) $$MCC=\frac{TP\times TN-FP\times FN}{\sqrt{\left(TP+FP)(TP+FN)(TN+FP)(TN+FN\right)}}$$

### 2.2. Performance Comparison with Previous Approaches

**Figure 1:**
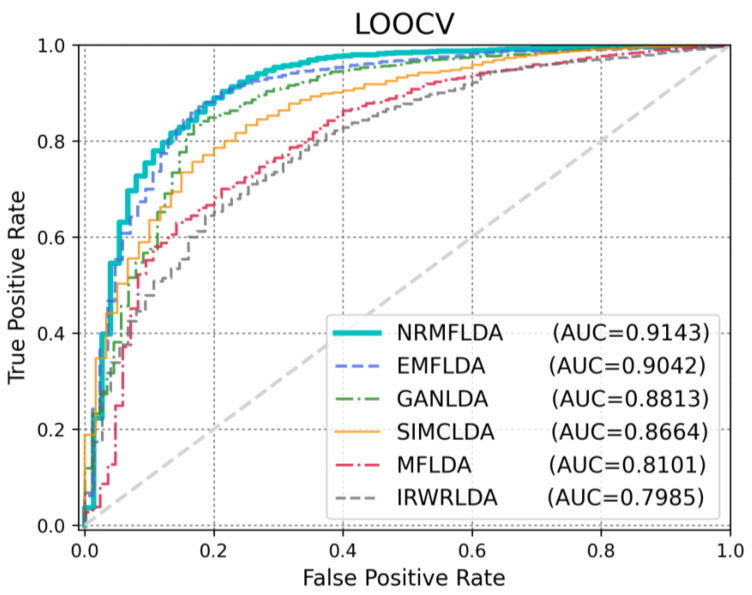
Performance validation of existing studies using LOOCV: NRMFLDA achieves superior AUC. The comparison of various methods using LOOCV highlights that NRMFLDA outperforms other models with an AUC of 0.9143. Among the compared models, NRMFLDA shows a clear advantage over EMFLDA, GANLDA, SIMCLDA, MFLDA and IRWRLDA, demonstrating its superior ability to capture discriminative features and improve classification performance.

**Figure 2:**
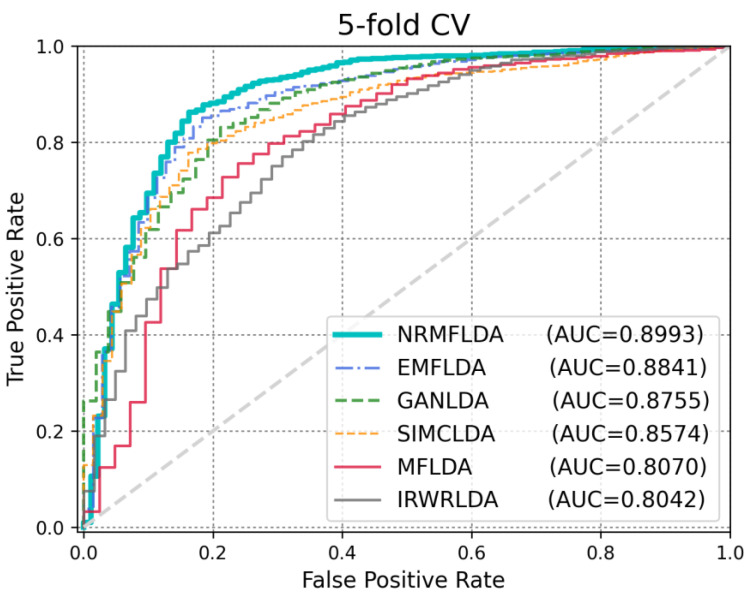
Performance validation of existing studies using 5-fold CV: NRMFLDA achieves superior AUC. The five-fold cross-validation results demonstrate that NRMFLDA achieves the highest AUC of 0.8993, outperforming other models such as EMFLDA, GANLDA, SIMCLDA, MFLDA and IRWRLDA. This validates the effectiveness of NRMFLDA in capturing relevant features and maintaining strong performance across different cross-validation settings.

The primary criterion for assessing the effectiveness of the proposed model lies in its ability to accurately identify disease-associated lncRNAs. To evaluate its performance, we conducted comparative experiments against five existing approaches—IRWRLDA [22], SIMCLDA [37], MFLDA [38], GANLDA [40] and EMFLDA [42]—using both leave-one-out cross-validation (LOOCV) and five-fold cross-validation (5-fold CV). As depicted in Figure 1, the results demonstrated that NRMFLDA achieved the highest performance with an AUC score of 0.9143 under the LOOCV framework, outperforming the other methods (EMFLDA: 0.9006, GANLDA: 0.8778, SIMCLDA: 0.8534, MFLDA: 0.7842, IRWRLDA: 0.7688). This finding highlights the superior predictive capability of NRMFLDA in comparison with previously established models. Additionally, to further validate the robustness of NRMFLDA, we applied 5-fold CV to evaluate its effectiveness in detecting lncRNA–disease associations. As shown in Figure 2, the proposed model exhibited an AUC value of 0.8993, once again surpassing the performance of competing methods. Moreover, Table 1 presents a detailed comparison based on multiple evaluation metrics, including accuracy (ACC), the Matthews correlation coefficient (MCC) and the area under the precision–recall curve (AUPRC). These metrics collectively reaffirm the superior performance of NRMFLDA, providing comprehensive evidence of its reliability and applicability in identifying lncRNA–disease associations.

### 2.3. Ablation Analysis

An ablation study is a valuable method used to systematically analyze the contribution of individual components or features within a model. By progressively removing or altering certain elements and observing the impact on performance, it allows for a deeper understanding of how each part influences the overall effectiveness of the model or algorithm. This approach is particularly useful in identifying critical factors that enhance model performance and in optimizing complex systems.

**Figure 3:**
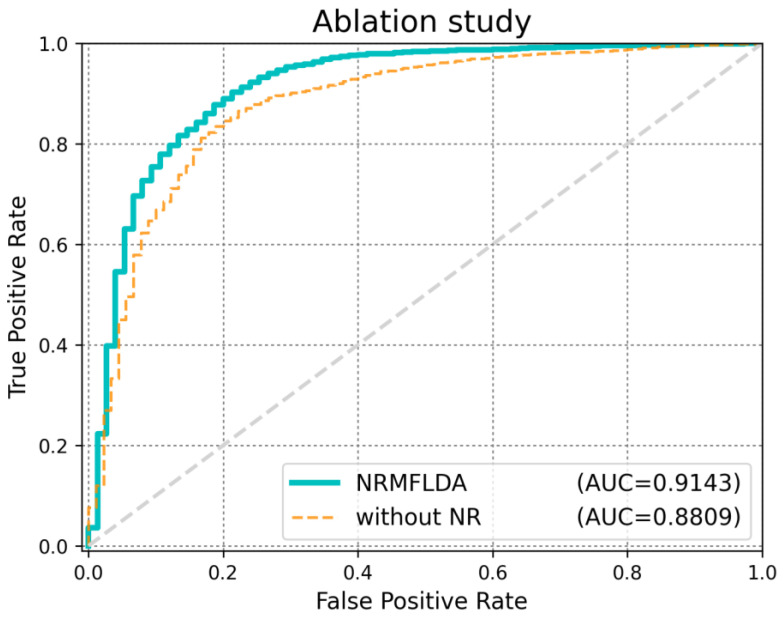
Ablation analysis: NRMFLDA with disease neighborhood regularization achieved better performance. The results from the ablation analysis indicate that incorporating disease neighborhood regularization (NR) in the NRMFLDA model significantly enhances its performance. By leveraging disease-specific feature relationships, the model demonstrates improved AUC, accuracy and MCC, highlighting the importance of disease neighborhood regularization in boosting model effectiveness.

In this study, we applied an ablation analysis to examine the role of disease neighborhood regularization within the NRMFLDA model, which is designed to improve the matrix factorization approach for identifying disease-related lncRNAs. The key innovation of NRMFLDA is its integration of disease neighborhood regularization, which aims to refine the identification process by leveraging shared characteristics between diseases. To evaluate the impact of this disease neighborhood regularization, we compared two variations of the NRMFLDA model: (1) the full NRMFLDA model that incorporates the disease neighborhood regularization (NR) and (2) a version of NRMFLDA where this disease neighborhood regularization (NR) was removed. As illustrated in Figure 3, the version of NRMFLDA that included neighborhood regularization outperformed the version without it, achieving an AUC score of 0.9143. This improvement highlights the crucial role of disease similarity in enhancing the model’s predictive accuracy. By considering the relationships between diseases, the model is able to refine its predictions and identify disease-related lncRNAs with greater performance. Table 2 also demonstrates that the proposed method achieves superior performance across various statistical metrics.

### 2.4. Case Studies

To evaluate the effectiveness of the proposed model in identifying disease-associated lncRNAs, we conducted case studies on three prominent human cancers: gastric, lung and prostate. By utilizing matrix factorization techniques, we captured the latent feature spaces of lncRNAs and diseases. The interaction between these latent spaces quantifies the association between lncRNA *i* and disease *u*. A higher inner product value indicates a stronger potential relationship between the two. Subsequently, the model was employed to rank the top 15 lncRNA candidates for each disease based on the scores derived from the NRMFLDA framework. These scores reflect the likelihood of association between specific lncRNAs and the target diseases. To validate the relevance of the predictions, the ranked lncRNAs were cross-referenced with two authoritative datasets, Lnc2Cancer v3.0 and lncRNADisease v2.0, which serve as gold standards in this field. This approach not only highlights the ability of NRMFLDA to prioritize meaningful lncRNA–disease associations but also emphasizes the utility of integrating matrix factorization in biomedical applications. The results provide a robust basis for further experimental validation and exploration of disease mechanisms involving lncRNAs.

Gastric cancer is a distinct form of malignant tumor that significantly contributes to cancer-related mortality worldwide [43]. Through extensive experimental studies, it has been demonstrated that certain lncRNAs play a critical role in influencing gastric cancer progression by regulating oncogenes [44]. Several lncRNAs, including GAPLIN, GClnc1, HOTAIR, H19 and MEG, have been identified as being strongly associated with gastric cancer [45,46]. Building upon these findings, we applied the NRMFLDA model to prioritize the top 15 lncRNAs most closely linked to gastric cancer based on the disease-related scores calculated by the framework. The prioritization process enabled us to highlight key candidates for further investigation. Notably, all 15 lncRNAs ranked at the top were confirmed to have established associations with gastric cancer, as verified through existing research and summarized in Table 3. This analysis underscores the ability of NRMFLDA to reliably identify and rank lncRNAs that are highly relevant to gastric cancer. The results not only align with known biological evidence but also pave the way for deeper exploration into the mechanisms by which these lncRNAs influence cancer development and progression.

Lung cancer, strongly influenced by tobacco smoke exposure, remains one of the leading causes of cancer-related deaths globally [47]. Broadly, lung cancer is categorized into two main types: non-small-cell lung cancer (NSCLC) and small-cell lung cancer (SCLC). Extensive research has identified a variety of lncRNAs that are closely associated with the development and progression of lung cancer [48,49]. To assess the effectiveness of our model in extracting lung cancer biomarkers, we prioritized the top 15 lncRNAs associated with the disease. These candidates were ranked based on their relevance scores, as determined by our NRMFLDA framework. Through data-driven validation, it was confirmed that all 15 top-ranked lncRNAs had known associations with lung cancer, as detailed in Table 4. This result highlights the robustness of the model in identifying biologically meaningful lncRNA candidates for lung cancer. By effectively distinguishing these key lncRNAs, the study provides a strong foundation for future research into their roles as potential diagnostic markers or therapeutic targets in lung cancer.

Prostate cancer is one of the most common malignancies affecting men. Several lncRNAs, such as NEAT1, H19, PVT1 and PCAT29, have been identified as having direct or indirect roles in influencing the onset and progression of this disease [50,51,52,53,54]. Recognizing the importance of these associations, we utilized the NRMFLDA framework to predict lncRNAs related to prostate cancer. The analysis prioritized the top 15 lncRNA candidates based on their disease-related scores (Table 5). Remarkably, all of these top-ranked candidates were verified to have established links to prostate cancer, reinforcing the reliability of our model’s predictions. This validation process further emphasizes the model’s ability to identify meaningful biomarkers. When the findings from all case studies were considered collectively, it became evident that the proposed NRMFLDA model excels at extracting disease-specific biomarkers. This strong performance underscores its potential as a valuable tool for uncovering lncRNA–disease relationships, which can drive future research into targeted diagnostics and therapies for prostate cancer and beyond.

## 3. Discussion

As medical technologies continue to evolve, human life expectancy has seen a steady increase. This shift is steering the society toward a new paradigm, where the emphasis extends beyond merely living longer to ensuring sustained health and quality of life throughout the aging process. In this context, the integration of preventive healthcare, personalized medicine and advanced diagnostics plays a critical role in addressing age-related challenges and fostering overall well-being across the lifespan. In this context, research on disease biomarker extraction has become crucial for understanding the mechanisms behind disease onset and progression. Long non-coding RNAs (lncRNAs) have been widely recognized for their pivotal roles in disease pathogenesis. They are involved in various biological processes, including cellular differentiation, immune response regulation, transcriptional and translational control and cell proliferation. Numerous studies have highlighted their importance in these mechanisms. Identifying novel associations between lncRNAs and diseases presents a significant opportunity to uncover the complex pathogenesis of human diseases. By deepening our understanding of these connections, researchers can pave the way for developing targeted diagnostics and therapies, ultimately contributing to better health outcomes in the era of extended life expectancy. For these reasons, numerous computational models have been proposed to elucidate the relationships between lncRNAs and various diseases.

This study introduces a new method, the neighborhood-regularized matrix factorization (NRMFLDA), designed to predict associations between lncRNAs and diseases. Matrix factorization (MF) is a prominent machine-learning technique commonly employed in recommendation systems due to its ability to identify hidden patterns in data. By integrating neighborhood regularization into the MF framework, NRMFLDA improves the prediction performance by leveraging prior knowledge about biological similarities. This approach offers a more reliable tool for uncovering the intricate relationships between lncRNAs and diseases, contributing to advancements in understanding complex molecular interactions and potential therapeutic targets. In NRMFLDA, we utilized both lncRNA expression profiles and disease neighborhood regularization to predict potential lncRNA–disease associations. Disease neighborhood regularization ensures that the model considers known disease-related factors, thereby improving the performance and relevance of the predictions. The implicit feedback model was applied by treating lncRNA expression values as part of the matrix factorization process. Notably, entries marked with zero in the matrix do not imply a lack of relationship but suggest that the potential association has yet to be uncovered, indicating areas where further exploration is needed. Through extensive validation, NRMFLDA demonstrated outstanding performance, achieving AUC scores of 0.9143 and 0.8993 in leave-one-out and five-fold cross-validation, respectively, outperforming previous models. These results highlight the model’s effectiveness in accurately predicting disease-related lncRNAs, surpassing traditional methods by leveraging neighborhood regularization to enhance predictive accuracy. We believe that NRMFLDA will serve as a valuable tool for discovering novel lncRNA–disease associations, offering significant promise in advancing disease biomarker identification. By integrating disease neighborhood regularization and similarity-based learning, this model could greatly contribute to the development of diagnostic, prognostic and therapeutic strategies for various human diseases, providing a foundation for targeted clinical applications.

## 4. Materials and Methods

### 4.1. Human lncRNA–Disease Association Data

We curated lncRNA–disease association data from publicly available online repositories. Specifically, the lncRNADisease v2.0 database provides 10,564 experimentally validated associations involving 19,166 lncRNAs and 529 diseases [55], while lnc2Cancer v3.0 offers 9254 associations between 2659 lncRNAs and 216 cancer subtypes [56]. To construct a high-confidence benchmark dataset, we consolidated the data from these two sources and removed duplicate entries to ensure the uniqueness of each lncRNA–disease pair. Based on the resulting dataset, we constructed a binary lncRNA–disease association matrix $R\in R^{N_{l}\times N_{d}}$ for use in matrix factorization, where a value of one indicates a confirmed association between a specific lncRNA and a disease. Here, $N_{l}$ and $N_{d}$ denote the total number of unique lncRNAs and diseases, respectively.

### 4.2. lncRNA Expression Data

Recent advances in high-throughput technologies have facilitated the generation of a wide range of biological datasets. Among these, omics data provide crucial insights into the regulatory mechanisms of lncRNA–related processes, including their involvement in disease progression. To enhance the accuracy of lncRNA–disease association predictions, we utilized lncRNA expression levels as a weight within the original association matrix. It is important to emphasize that a zero value in the lncRNA–disease matrix does not imply the complete absence of an association; rather, it indicates that the relationship has yet to be identified, even though it may exist. In this context, we leveraged lncRNA expression profiles to estimate potential associations between lncRNAs and diseases, even in the absence of direct associations in the dataset. The expression profiles were obtained from the UCSC Genome Bioinformatics database (http://genome.ucsc.edu/, accessed on 16 January 2025) and subsequently standardized using min–max normalization to facilitate downstream analysis. This approach allows for a more comprehensive understanding of the potential lncRNA–disease interactions that have not yet been experimentally confirmed.

### 4.3. Disease Semantic Similarity

To measure the degree of similarity between diseases, we utilized a directed acyclic graph (DAG), which is a unique type of graph that consists of directed connections and prohibits the formation of cycles. This structure is especially well suited for representing hierarchical relationships, making it an ideal choice for analyzing disease ontologies and their complex organization. In our approach, the disease DAG corresponding to a node P is defined as (P,A(P),EG(P)). Here, A(P) represents the collection of all ancestor nodes of P, capturing its hierarchical structure within the graph. On the other hand, EG(P) denotes the set of edges linking each parent node to its associated child nodes, thereby illustrating the relational connections that underpin the DAG framework. This representation ensures a comprehensive understanding of the hierarchical and relational dynamics within the disease ontology. These relationships are formalized through Equations (7) and (8), which rigorously define the structural dynamics within the DAG. By leveraging these equations, we facilitate the computation of disease similarity, integrating both topological and contextual information inherent in the graph structure.

(7) $$DV\left(P\right)=\sum_{c\in A\left(P\right)}P_{P}\left(c\right)$$

(8) $$\left\{\begin{array}{c} P_{P}(c)=1\;\;\;\;\;\;\;\;\;\;\;\;\;\;\;\;\;\;\;\;\;\;\;\;\;\;\;\;\;\;\;\;\;\;\;\;\;\;\;\;\;\;\;\;\;\;\;\;\;\;\;\;\;\;if\;c=P\\ P_{P}(c)=\max\{\Delta_{*}P_{P}(c^{\prime })|c^{\prime }\in children\;of\;c\}\;if\;c\neq P \end{array}\right.$$

In the presented equations, the semantic contribution factor (Δ) serves as a key component, quantifying the increase in semantic association between two diseases as their proximity within the semantic structure becomes closer. This concept relies on the principle that diseases located nearer to each other in a directed acyclic graph (DAG) are more likely to exhibit shared characteristics. Based on this assumption, the scoring model assesses the degree of commonality within the DAG, implying that a higher degree of overlap corresponds to greater similarity. To enable this assessment, the disease semantic similarity matrix (S) is introduced as a systematic tool for quantifying pairwise disease similarity. Specifically, Equation (9) defines the semantic similarity between diseases *i* and *j*, providing a robust and scalable framework for exploring disease relationships through their semantic context and hierarchical positioning.

(9) $$SS(d(i),d(j))=\frac{\sum_{t\in A(i)\cap A(j)}\left(P_{i}(t)+P_{j}(t)\right)}{DV(i)+DV(j)}$$

### 4.4. Gaussian Interaction Profile Kernel

The Gaussian interaction profile (GIP) kernel has been extensively employed across various fields to effectively model interaction patterns, including associations between genes, diseases and even social network users [57,58]. Due to its proven reliability, we applied the GIP kernel to compute similarity scores between lncRNAs and diseases, leveraging known lncRNA–disease association data. In this framework, IP(*d*(*i*)) represents a profile vector that indicates whether a specific disease is associated with a given lncRNA *l*(*i*). This profile serves as a structured depiction of lncRNA–disease interactions, facilitating the calculation of GIP-based similarity between any two diseases, *d*(*i*) and *d*(*j*), using the following mathematical formulation. This approach not only ensures precision in similarity estimation but also provides a robust foundation for analyzing complex interaction networks.


(10) $$GS\left(m(i),\;m(j))=\exp(-r_{l}\;∥\;IP(m(i)\right)-IP\left(m\left(j\right)\right){∥}^{2})$$

In this framework, GS refers to the Gaussian interaction profile (GIP) kernel similarity, and $r_{m}$ serves as a hyperparameter that regulates the kernel’s bandwidth. Building on findings from previous studies, we set $r_{m}^{\prime }$ to 1 as the default value. This choice ensures consistency and simplifies the parameter selection process. By employing this configuration, we computed similarity scores between diseases using the GIP methodology, providing a robust and scalable approach for quantifying relationships within the data.

(11) $$r_{l}=\frac{r_{d}^{\prime }}{\frac{1}{n_{l}}\sum_{i=1}^{n_{d}}||IP(m(i))|{|}^{2}}$$

### 4.5. Comprehensive Disease Similarity in Disease Network

To construct the disease similarity network, we first calculated an integrated similarity score between diseases. This score combines two major factors: the semantic similarity of diseases (SS) and the Gaussian interaction kernel similarity of diseases (GS). By merging these two metrics, we obtained a unified weight value, which serves as the edge weight in the disease similarity network (DS). The relationship between these components can be expressed through the following equation:

(12) $$DS\left(d\left(i\right),d\left(j\right)\right)=\left\{\begin{array}{c} SS\left(d\left(i\right),d\left(j\right)\right)\;if\;d\left(i\right)\;and\;d\left(j\right)\;has\;semantic\;similarity\\ GS\left(d\left(i\right),d\left(j\right)\right)\;otherwise \end{array}\right.$$

### 4.6. Similarity Constrained Matrix Factorization

**Figure 4:**
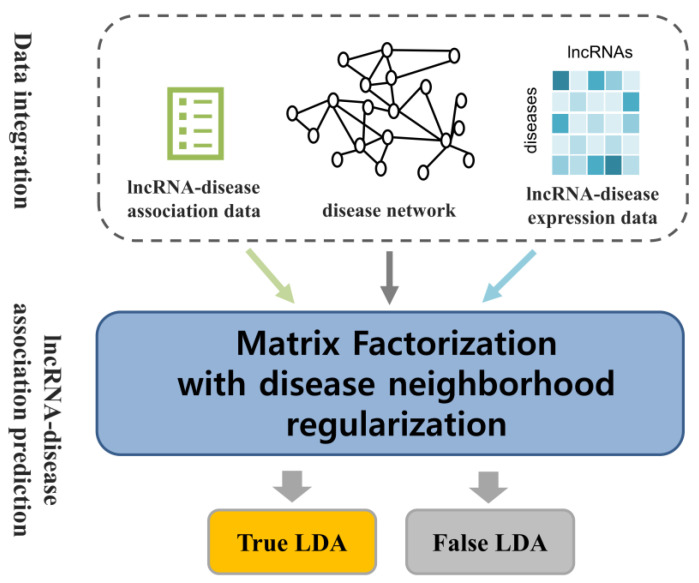
Workflow of NRMFLDA. The NRMFLDA framework combines matrix factorization with disease-specific neighborhood regularization to predict novel lncRNA–disease associations. By transforming known associations into a unified latent space and incorporating lncRNA expression profiles, NRMFLDA enhances the precision of these predictions and aligns computational results with biological mechanisms.

Matrix factorization techniques have achieved remarkable results in recommendation systems [38]. Nevertheless, the effectiveness of these models often diminishes when applied to large-scale, sparse datasets, as is common with the original interaction matrix. Specifically, matrix-factorization-based approaches encounter significant challenges, such as the cold start problem, particularly when certain lncRNAs have limited disease associations within the binary adjacency matrix. To address these limitations, several advanced matrix factorization techniques and machine-learning models have been developed, leveraging diverse biological datasets to improve performance [59,60]. In this study, we incorporate disease network information as auxiliary data to enhance predictive accuracy (Figure 4). The disease network is represented as a graph, where each node corresponds to a specific disease, and the edges denote the similarity weights between pairs of diseases. In this context, the similarity weight $S_{u,v}$ encapsulates the extent to which disease $D_{u}$ resembles disease $D_{v}$. These weights provide a quantitative measure of similarity, reflecting biological relationships or semantic similarities between diseases. By incorporating this network structure, valuable auxiliary information can be utilized to address sparsity issues and improve model performance in downstream prediction tasks. When the network influence is applied, the properties of each disease are shaped by the characteristics of its immediate neighbors, represented by $E_{u}$. This approach is based on the concept that nodes exhibiting similar structural functions within the network tend to be positioned near one another. As a result, the latent feature vector of disease D is largely influenced by the latent feature vectors of its neighboring nodes $v\in E_{u}$. The predicted latent feature vector ${\hat{D}}_{u}$ is derived from the feature vectors of its direct neighbors. The mathematical formulation is presented as

(13) $${\hat{D}}_{u}\frac{\sum_{v\in E_{u}}S_{u,v}D_{v}}{\sum_{v\in E_{u}}S_{u,v}}=\frac{\sum_{v\in E_{u}}S_{u,v}D_{v}}{\left|E_{u}\right|}$$

By leveraging the intrinsic properties of diseases within the disease similarity network, the latent feature vector for a disease can be redefined. Specifically, it is computed as a weighted average of the latent feature vectors of directly connected diseases. This approach ensures that the similarity-based relationships are effectively incorporated into the estimation process, thereby enhancing the representation of the disease in the latent space.

(14) $$\left(\begin{array}{c} {\hat{D}}_{u,1}\\ {\hat{D}}_{u,2}\\ \ldots \\ {\hat{D}}_{u,k} \end{array}\right)\left(\begin{array}{cccc} D_{1,1} & D_{2,1} & \ldots & D_{N,1}\\ D_{1,2} & D_{2,2} & \ldots & D_{N,2}\\ \ldots & \ldots & \ldots & \ldots \\ D_{1,k} & D_{2,k} & \ldots & D_{N,k} \end{array}\right)\left(\begin{array}{c} S_{u,1}\\ S_{u,2}\\ \ldots \\ S_{u,N} \end{array}\right)$$

The consideration of the disease similarity network as implicit feedback does not alter the conditional distribution of the established disease–lncRNA associations. Instead, it primarily focuses on incorporating the latent feature vectors of diseases. Consequently, the conditional probability can be reformulated and expressed in the following manner, maintaining consistency with the original distributional assumptions.

(15) $$p(R|L,D,\;\sigma_{R}^{2})=\prod_{u=1}^{N_{l}}\prod_{i=1}^{N_{d}}{\left[N(R_{u,i}|g\left(L_{u}^{T}D_{i}\right),\sigma_{R}^{2})\right]}^{I_{u,i}^{R}}$$

To mitigate the risk of overfitting, a zero-mean Gaussian prior is applied to the latent vectors of diseases. Inspired by the observation that the characteristics of a disease are significantly influenced by its immediate neighbors, the conditional distribution of a disease’s latent vector is defined based on the latent vectors of its directly connected neighbors, as described below.

(16) $$p(L,D|R,S,\sigma_{R}^{2},\sigma_{S}^{2},\sigma_{L}^{2},\sigma_{D}^{2})\propto p(R|D,L,\sigma_{R}^{2})p(D|S,\sigma_{D}^{2},\sigma_{S}^{2})p(L|\sigma_{L}^{2})\;\;=\;\prod_{u=1}^{N_{d}}\prod_{i=1}^{N_{l}}{\left[N(R_{u,i}|g\left(D_{u}^{T}L_{i}\right),\sigma_{R}^{2})\right]}^{I_{u,i}^{R}}\;\times \prod_{u=1}^{N_{d}}N(D_{u}|\sum_{v\in E_{u}}S_{u,v}D_{v},\sigma_{S}^{2}I)\;\times \prod_{u=1}^{N_{d}}N(D_{u}|0,\sigma_{D}^{2}I)\times \;\prod_{i=1}^{N_{l}}N(L_{i}|0,\sigma_{L}^{2}I)$$

The log posterior probability is derived, aiming to identify the most likely latent vectors for lncRNAs $L_{i}$ and diseases $D_{u}$. The objective is to ensure that the inner product of these latent vectors closely approximates the corresponding entries in the binary association matrix $R_{u,i}$. To refine the cost function and improve its accuracy, additional terms related to lncRNAs were introduced. These terms enhance the representation of the latent vector $D_{u}$ by naturally integrating the characteristics of neighboring diseases $D_{v}$ within the disease similarity network S. Furthermore, we define the lncRNA expression weight matrix W, which facilitates efficient training of the latent vectors for both lncRNAs and diseases.

(17) $$lnp(L,D|R,S,\sigma_{R}^{2},\sigma_{S}^{2},\sigma_{M}^{2},\sigma_{D}^{2})=\;-\frac{1}{2\sigma_{R}^{2}}\sum_{u=1}^{N_{d}}\sum_{i=1}^{N_{l}}W_{u,i}{\left(R_{u,i}-g\left(D_{u}^{T}L_{i}\right)\right)}^{2}\;-\frac{1}{2\sigma_{D}^{2}}\sum_{u=1}^{N_{d}}D_{u}^{T}D_{u}-\frac{1}{2\sigma_{L}^{2}}\sum_{i=1}^{N_{l}}L_{i}^{T}L_{i}\;-\frac{1}{2\sigma_{S}^{2}}\sum_{u=1}^{N_{d}}\left({\left(D_{u}-\sum_{v\in E_{u}}S_{u,v}D_{v}\right)}^{T}\left(D_{u}-\sum_{v\in E_{u}}S_{u,v}D_{v}\right)\right)$$

**Figure 5:**
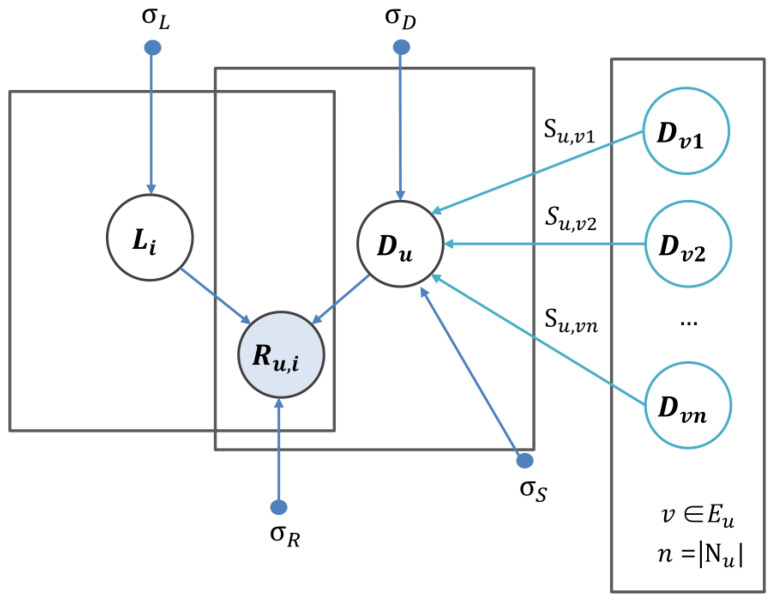
Graphical modeling of disease neighborhood regularization.

Optimizing the log posterior with respect to the latent vectors of lncRNAs and diseases can be interpreted as minimizing the corresponding cost function described below (Equation (10)). The primary objective is to reduce the discrepancy between the entries in the binary association matrix $R_{u,i}$ and the dot product of the latent vector of the lncRNA $L_{i}$ and that of the disease $D_{u}$. This approach ensures that the latent representations accurately capture the observed associations while maintaining computational efficiency. The effect of disease neighborhood regularization is illustrated in Figure 5.

(18) $$L(R,S,L,D)=\frac{1}{2}\sum_{u=1}^{N_{d}}\sum_{i=1}^{N_{l}}W_{u,i}{\left(R_{u,i}-g\left(D_{u}^{T}L_{i}\right)\right)}^{2}\;+\frac{\lambda_{D}}{2}\sum_{u=1}^{N_{d}}D_{u}^{T}D_{u}+\frac{\lambda_{L}}{2}\sum_{i=1}^{N_{l}}L_{i}^{T}L_{i}\;+\frac{\lambda_{S}}{2}\sum_{i=1}^{N_{d}}{(\left(D_{u}-\sum_{v\in E_{u}}S_{u,v}D_{u}\right)}^{T}\left(D_{u}-\sum_{v\in E_{u}}S_{u,v}D_{u})\right)$$

## 5. Conclusions

This study presents neighborhood regularization matrix factorization (NRMFLDA), a novel approach designed to predict lncRNA–disease associations. By integrating matrix factorization with disease neighborhood regularization, NRMFLDA improves prediction accuracy, offering a more reliable framework for uncovering the complex relationships between lncRNAs and diseases. The results of our extensive validation, which show AUC scores of 0.9143 in leave-one-out cross-validation and 0.8993 in five-fold cross-validation, demonstrate the superiority of NRMFLDA over traditional models. These findings highlight the model’s potential in advancing disease biomarker discovery and its capacity to provide valuable insights into the molecular mechanisms underlying human diseases. The significance of this research lies in its potential to accelerate the identification of novel lncRNA–disease associations, which can lead to the development of more precise diagnostic tools and therapeutic strategies. As the world transitions toward an era of personalized medicine and extended life expectancy, such predictive models will be critical in addressing age-related health challenges and improving overall well-being. By offering a more targeted and effective approach to understanding disease mechanisms, NRMFLDA holds promise for revolutionizing disease biomarker identification, which could play a pivotal role in the prevention, diagnosis and treatment of various diseases.

Future research should aim to refine the NRMFLDA model by incorporating additional layers of biological information, such as protein–protein interactions and genetic variations, to further enhance its predictive performance. In particular, integrating features related to target genes—such as gene expression, DNA methylation and somatic mutation data—could provide a more comprehensive view of lncRNA–disease associations. However, accurately predicting disease-related lncRNAs remains challenging due to two key factors: the limited availability of experimentally validated associations and the complex, often poorly understood regulatory mechanisms involving lncRNAs. These challenges hinder the construction of reliable training datasets and complicate the design of biologically meaningful inference models. These multi-omics features may help capture complex regulatory mechanisms that are not addressed in the current framework. The continued advancement of computational models is expected to drive progress in bioinformatics and personalized medicine, ultimately establishing a solid foundation for improving human health in the future.

## Figures and Tables

**Table 1:** Comprehensive performance comparison using global LOOCV based on various metrics.

Method	AUC (LOOCV)	AUC (5-Fold CV)	AUPRC	ACC	MCC
NRMFLDA	0.9143	0.8993	0.3451	0.9013	0.7727
EMFLDA	0.9042	0.8841	0.2068	0.8793	0.8912
GANLDA	0.8813	0.8755	0.1572	0.8735	0.8671
SIMCLDA	0.8664	0.8574	0.0301	0.8481	0.8474
MFLDA	0.8101	0.8070	0.0138	0.8024	0.8016
IRWRLDA	0.7985	0.8042	0.0043	0.7618	0.7602

**Table 2:** Comprehensive performance comparison using global LOOCV based on various metrics.

Method	AUC (LOOCV)	AUC (5-Fold CV)	AUPRC	ACC	MCC
NRMFLDA	0.9143	0.8993	0.3451	0.9013	0.7727
NRMFLDA without NR	0.8809	0.8041	0.1472	0.8243	0.7361

**Table 3:** Top 15 gastric-cancer-related lncRNA candidates.

Rank	LncRNA	Evidence
1	RP11-167N4	lnc2Cancer
2	MYLK-AS1	lnc2Cancer
3	MAFG-AS1	lnc2Cancer
4	LINC01071	lnc2Cancer
5	AK001058	lnc2Cancer
6	LINC00673	lnc2Cancer
7	CCAT2	lnc2Cancer
8	GIHCG	lnc2Cancer
9	LINP1	lnc2Cancer
10	ZXF2	lnc2Cancer
11	RRP1B	lncRNADisease
12	GCAWKR	lnc2Cancer
13	EPEL	lnc2Cancer
14	LINC02407	lnc2Cancer
15	LINC00086	lnc2Cancer

**Table 4:** Top 15 lung-cancer-related lncRNA candidates.

Rank	LncRNA	Evidence
1	TCF7	lncRNADisease
2	SPRY4-IT1	lncRNADisease
3	LINC01186	lnc2Cancer, lncRNADisease
4	LUCAT1	lncRNADisease
5	PCAT6	lnc2Cancer, lncRNADisease
6	LCAL1	lnc2Cancer, lncRNADisease
7	LSINCT3	lnc2Cancer, lncRNADisease
8	BANCR	lnc2Cancer, lncRNADisease
9	H19	lnc2Cancer, lncRNADisease
10	GAS5	lnc2Cancer, lncRNADisease
11	CASC8	lncRNADisease
12	RIOX2	lncRNADisease
13	PVT1-5	lnc2Cancer, lncRNADisease
14	MEG3	lnc2Cancer, lncRNADisease
15	CCAT2	lnc2Cancer, lncRNADisease

**Table 5:** Top 15 prostate-cancer-related lncRNA candidates.

Rank	LncRNA	Evidence
1	SPRY4-IT1	lnc2Cancer, lncRNADisease
2	PCGEM1	lnc2Cancer, lncRNADisease
3	PCA3	lnc2Cancer, lncRNADisease
4	SNHG1	lnc2Cancer, lncRNADisease
5	PCAT2	lnc2Cancer, lncRNADisease
6	HOTAIR	lnc2Cancer, lncRNADisease
7	ATB	lncRNADisease
8	UCA1	lnc2Cancer, lncRNADisease
9	SNHG5	lnc2Cancer, lncRNADisease
10	MEG3	lnc2Cancer, lncRNADisease
11	PRNCR1	lnc2Cancer, lncRNADisease
12	CBR3-AS1	lncRNADisease
13	FALEC	lnc2Cancer, lncRNADisease
14	DRAIC	lnc2Cancer, lncRNADisease
15	PCAT1	lnc2Cancer, lncRNADisease

## Data Availability

All the relevant data are included within the paper.
